# Comprehensive economic evaluation of thermotherapy for the treatment of cutaneous leishmaniasis in Colombia

**DOI:** 10.1186/s12889-018-5060-2

**Published:** 2018-01-29

**Authors:** Jaiberth Antonio Cardona-Arias, Liliana López-Carvajal, Mery Patricia Tamayo-Plata, Iván Darío Vélez

**Affiliations:** 1 Universidad de Antioquia, Universidad Cooperativa de Colombia, Medellin, Colombia; 2 PECET Program for the Study and Control of Tropical Diseases, University of Antioquia, Medellin, Colombia; 3 School of Economics and Finance, EAFIT University, Medellin, Colombia

**Keywords:** Cost-effectiveness evaluation, Cutaneous leishmaniasis, Thermotherapy, Sodium antimony gluconate, Colombia

## Abstract

**Background:**

Cutaneous leishmaniasis causes a high disease burden in Colombia, and available treatments present systemic toxicity, low patient compliance, contraindications, and high costs. The purpose of this study was to estimate the cost-effectiveness of thermotherapy versus Glucantime in patients with cutaneous leishmaniasis in Colombia.

**Methods:**

Cost-effectiveness study from an institutional perspective in 8133 incident cases. Data on therapeutic efficacy and safety were included, calculating standard costs; the outcomes were disability adjusted life years (DALYs) and the number of patients cured. The information sources were the Colombian Public Health Surveillance System, disease burden studies, and one meta-analysis of controlled clinical trials. Incremental cost-effectiveness was determined, and uncertainty was evaluated with tornado diagrams and Monte Carlo simulations.

**Results:**

Thermotherapy would generate costs of US$ 501,621; the handling of adverse effects, US$ 29,224; and therapeutic failures, US$ 300,053. For Glucantime, these costs would be US$ 2,731,276, US$ 58,254, and US$ 406,298, respectively. With thermotherapy, the cost would be US$ 2062 per DALY averted and US$ 69 per patient cured; with Glucantime, the cost would be US$ 4241 per DALY averted and US$ 85 per patient cured. In Monte Carlo simulations, thermotherapy was the dominant strategy for DALYs averted in 67.9% of cases and highly cost-effective for patients cured in 72%.

**Conclusion:**

In Colombia, thermotherapy can be included as a cost-effective strategy for the management of cutaneous leishmaniasis. Its incorporation into clinical practice guidelines could represent savings of approximately US$ 10,488 per DALY averted and costs of US$ 116 per additional patient cured, compared to the use of Glucantime. These findings show the relevance of the incorporation of this treatment in our country and others with similar parasitological, clinical, and epidemiological patterns.

## Background

Leishmaniasis is a major public health disease – particularly in the vector-borne group – given that it is endemic in 98 countries and approximately 350 million people are at risk worldwide. According to the World Health Organization (WHO), its incidence ranges from 200,000 to 400,000 cases for visceral leishmaniasis and 700,000 to 1200,000 for cutaneous leishmaniasis. The latter is distributed worldwide, predominating in the Americas, the Mediterranean region, and Asia. Approximately 70%–75% of the global incidence occurs in 10 countries: Colombia, Afghanistan, Algeria, Brazil, Costa Rica, Ethiopia, Iran, Peru, Sudan, and Syria [2, 27].

The disease presents a variety of forms, varying in severity from a cutaneous ulcer that heals spontaneously to a visceral disease. There are three main clinical forms of the disease: i) cutaneous (localized, and disseminated or diffuse), ii) mucocutaneous, and iii) visceral or kala-azar. The form is determined by the species of the infectious parasite and the immune status of the host. In general terms, it mainly affects the poorest populations, being associated with poor housing conditions and a lack of economic resources [6, 7, 21].

Although there are effective therapies for cutaneous leishmaniasis, first-line treatments (pentavalent antimonials) have limitations due to safety concerns and their cost-effectiveness, causing adverse effects such as arthralgia, myalgia, respiratory disorders, and systemic toxicity. Adding to these concerns, these treatments are contraindicated in multiple groups of people such as pregnant women, and the cost of these drugs in Colombia is very high. These limitations have led to the need to investigate alternative treatments such as thermotherapy, which has shown favorable efficacy and safety results [4, 13].

Regarding this therapy, one meta-analysis of eight clinical trials evaluated thermotherapy in 622 patients and showed an efficacy of 73.2% (95% CI = 69.6–76.7); this meta-analysis compared thermotherapy to the use of pentavalent antimonials in 667 infected patients and found a similar statistical efficacy (70.6% CI 95% = 67.1–74.1). In this meta-analysis, there was a high heterogeneity in the findings, methodology of the studies, infecting species, and other parasitological and clinical characteristics that prevent the extrapolation of these results to Colombia [4].

Globally, despite the great number of studies on cutaneous leishmaniasis, the papers are mainly focused on clinical and preclinical aspects, epidemiological research on the magnitude and risks of the disease, and drug efficacy; systematic reviews have also been published on the quality of clinical trials in this disease [16], and research on health economics is scarce. In this regard, there is one systematic review of economic evaluations available that identifies, describes, classifies, and analyzes the scientific evidence related to the economic evaluation of the health technologies used in visceral leishmaniasis, concluding that the most common studies are based on cost-effectiveness analysis [11].

In addition to the above, scarce economic evaluations of cutaneous leishmaniasis have been found in the world scientific literature, after conducting a search of studies published during the 1980–2014 period in the MEDLINE-PubMed, MEDLINE-Ovid, ScienceDirect, SciELO, Health Technology Assessment, and NHS-Economic Evaluation databases and using the following search strategies: *i*) economic evaluation &leishmania, *ii*) costs &leishmania, *iii*) cost-effectiveness &leishmania, *iv*) cost-utility &leishmania, and *v*) cost-benefit &leishmania. Based on the above search strategy, five economic evaluations were found. One was an incomplete economic evaluation, in which the costs of laboratory tests, treatment, and hospitalization were determined [5]. In Kabul, Afghanistan, a cost-effectiveness evaluation was developed for the intralesional and intramuscular administration of antimonials, concluding that the cost with the standard treatment was US$ 27 (95% CI = 20–36) per patient cured and US$ 1200 (95% CI = 761–1827) per disability adjusted life year (DALY) averted [20]. In Argentina, it was reported that the incremental cost-effectiveness ratio (ICER) was US$ 459 for early diagnosis per DALY averted and US$ 16,478 for insecticide prevention per DALY averted [17]. In Latin American countries, a simulation study on the cost-effectiveness of a potential vaccine for the disease was conducted [3]. Finally, a cost-effectiveness study of the standard treatment was conducted in Colombia in 2004 during an outbreak [26].

In addition to the previous studies, a cost-effectiveness study was conducted in Colombia in 2014 for first-line treatments for infected elderly people, concluding that the most cost-effective treatment is based on pentavalent antimonials, compared to miltefosine, pentamidine, and no treatment [15].

The above shows that, in Colombia and in the world literature in general, there is no comprehensive economic research comparing the cost-effectiveness of thermotherapy versus pentavalent antimonials in patients with cutaneous leishmaniasis. This research is extremely important to lead to a more efficient use of health system resources (particularly those destined for the control of vector diseases) and to generate evidence on the potential clinical, epidemiological, and economic benefits of incorporating this therapy into clinical practice guidelines and public health programs. This is even more important when we bear in mind that leishmaniasis represented 4.7% of the parasitic and vector-borne disease burden in the world in 2012, with 3,374,000 DALYs, and that in Colombia in 2000, it caused 1356 DALYs [28].

Consistent with the above, the aim of this study is to estimate the cost-effectiveness ratio of thermotherapy compared to Glucantime in patients with cutaneous leishmaniasis in Colombia.

## Methods

### Type of study

Cost effectiveness analysis from an institutional perspective, using a decision tree.

### Components of the PICO T-R question: Population, intervention, comparator, outcome; time, and resources

#### Population

Incident cases of cutaneous leishmaniasis diagnosed in Colombia during 2015, which totaled 8113 cases according to the National Public Health Surveillance System [8].

#### Intervention

Application of local heat (thermotherapy) by radiofrequency at 50 °C for 30 s three times a week with ThermoMed® equipment from Thermosurgery Inc. (Phoenix, Arizona, USA). The application of thermotherapy begins with the establishment of asepsis in the lesion and infiltration with 2% xylocaine local anesthesia. Then, thermotherapy is applied to the center and active edges of the lesions until they are completely treated. Once this phase is finished, fusidic acid is applied for 10 days [10].

#### Comparator

There are several pentavalent antimonials in the market, mainly sodium stibogluconate (Pentostam®) and meglumine antimoniate (Glucantime®), which are similar and only vary in antimony content [18]. In Colombia, Glucantime is the recommended first-line treatment.

#### Outcomes or definition of effectiveness

The primary outcome of effectiveness was DALYs averted, and the secondary measure of effectiveness was the number of patients cured. Cure was defined as the disappearance or re-epithelialization of the lesions, complete loss of induration or flattening of the lesion, and disappearance of lymphangitis or adenitis up to 3 months after the end of treatment, without reactivation of the lesion or the appearance of new lesions or mucosal involvement 6 months post treatment [18].

One DALY corresponds to 1 year of healthy life lost due to ill-health, disability or premature death, measuring the disease burden for specific causes. It provides a general and specific view of the health status of a population, helping to *"make decisions and comparisons, prioritize resources, and implement follow-up in the health system"* [19]. In its calculation, the following components are taken into account: i) life expectancy, which, according to the recommendation of previous studies of disease burden in Colombia, corresponds to 80 years for men and 82.5 for women; ii) the age weight, with which the years of healthy life of the young are valued more by having greater productive capacity; iii) the disability weight, which relates the specific disability of the disease during its course and the time lost by mortality, which is zero for ideal health states and one for states comparable to death; and (iv) the discount rate, which gives greater importance to current benefits, compared to future benefits [19].

To calculate DALYs, the years of life lost (YLL) due to premature death are combined with the years lived with disability (YLD) due to a disease with a given severity and duration, based on the following statistics quoted from the study by Martínez [12], which are simultaneously based on WHO reports [12, 14, 29]:

$$ \mathbf{YLL}=\mathrm{KCerA}/\left(\mathrm{r}+\upbeta \right)2\;\Big\{\mathrm{e}\hbox{-} \left(\mathrm{r}+\upbeta \right)\left(\mathrm{L}+\mathrm{A}\right)\left[\hbox{-} \left(\mathrm{r}+\upbeta \right)\left(\mathrm{L}+\mathrm{A}\right)\hbox{-} 1\right]\hbox{--} \mathrm{e}\hbox{-} \left(\mathrm{r}+\upbeta \right)\mathrm{A}\ \left[\hbox{-} \left(\mathrm{r}+\upbeta \right)\mathrm{A}\hbox{-} 1\left]\Big\}+\right[\left(1\hbox{-} \mathrm{K}\right)/\mathrm{r}\right]\left(1\hbox{-} \mathrm{e}\hbox{-} \mathrm{rL}\right) $$

$$ \mathbf{YLD}=\mathrm{DKCerAs}/\left(\mathrm{r}+\upbeta \right)\ 2\ \Big\{\mathrm{e}\hbox{-} \left(\mathrm{r}+\upbeta \right)\left(\mathrm{Ld}+\mathrm{As}\right)\left[\hbox{-} \left(\mathrm{r}+\upbeta \right)\left(\mathrm{Ld}+\mathrm{As}\right)\hbox{-} 1\right]\hbox{--} \mathrm{e}\hbox{-} \left(\mathrm{r}+\upbeta \right)\mathrm{As}\ \left[\hbox{-} \left(\mathrm{r}+\upbeta \right)\mathrm{As}\hbox{-} 1\left]\Big\}+\right[\left(1\hbox{-} \mathrm{K}\right)/\mathrm{r}\right]\ \left(1\hbox{-} \mathrm{e}\hbox{-} \mathrm{rLd}\right) $$

DALYs were estimated in an Excel spreadsheet from the WHO Global Burden of Disease (2016) study, with the following data taken from the National Public Health Surveillance System (Sistema Nacional de Vigilancia en Salud Pública–SIVIGILA) and studies on disease burden in Colombia in 2005 and 2010 [1, 8, 19]:

K = 0.5. Age-weighting factor (0 = no age weights; 1 = full age weights).

β = 0.04. Age-weighting parameter.

*r* = 0.03. Discount rate for the years of life by time preference.

C = 0.1658. Model constant, standard age weighting.

A: Age of the patient who died from leishmaniasis, corresponding to one woman in the age group between 70 and 74 years and four men in the age groups of 0–4, 30–34, 45–49, and over 80 years old.

L: Life expectancy. Men 80; women 82.5 years.

D: Disability weight. According to the WHO recommendation, D is 0.022875 for patients with cutaneous leishmaniasis of all age groups treated and untreated.

As: Age of the patient at the time of the disease, by estimating the percentage distribution by age group and sex of incident cases of cutaneous leishmaniasis in Colombia and the average age of each age group.

Ld: Duration of the event. According to the Colombian disease burden studies, for the age groups of 0–4, 5–14, 15–29, 30–44, 45–59, 60–69, 70–79, and over 80 years of age, the following durations are found: 1.2, 10.4, 23.2, 36.5, 51.1, 64.6, 74.6, and 91.7, respectively [1, 8, 19].

*Time horizon*: One year, corresponding to the time window in which incident cases of morbidity and mortality due to this disease were recorded. In addition, it is sufficient time for the evaluation of the outcomes of interest because DALYs take into account morbidity and mortality due to leishmaniasis in 1 year and cure is defined based on a follow-up of 6 months. Given this time horizon, no discount rate was applied.

*Resources*: A cost per patient expressed in US dollars was determined and validated by five experts (three doctors and two dermatologists who handle this type of patient) in the phases of identification, measurement, and evaluation. In the identification phase, the direct cost-generating events described in the management guides and by the experts were identified, including the costs of applying the intervention (medication, doctor, and nurse), the use of diagnostic aids, and the management of local and systemic side effects. In the measurement phase, the quantity and frequency of use of each identified cost were established. The evaluation phase included data from the standardized tariff manuals for Colombia such as Traffic Accident Mandatory Insurance (SOAT) and the Drug Pricing Information System (SISMED), although it should be noted that the monetary results do not correspond to costs but only to tariffs or rates that have been agreed upon for the provision of health services in Colombia.

### Sources of information

The number of incident cases was taken from official SIVIGILA records. The probabilities of cure and therapeutic failure as well as local or systemic adverse events were taken from a phase III controlled clinical trial conducted in Colombia to assess the efficacy of thermotherapy and from one meta-analysis that evaluated the therapeutic efficacy of thermotherapy in cutaneous Leishmaniasis [4, 10].

### Description of the decision model

A decision tree was constructed whose initial node explains the two alternatives compared and includes the following states:

1. Cure or therapeutic failure, the latter including persistence of the lesion, incomplete epithelization, induration, mucosal involvement, or recovery of the lesion 6 months after the end of treatment.
2. According to therapeutic safety data, the second condition includes cure without adverse effects or with local or systemic effects. Adverse events were classified according to the *"Common Terminology Criteria for Adverse Events (CTCAE) v.3"* into local events such as pain, burning, pruritus, erythema, edema, and inflammation at the site of administration and systemic events such as laboratory abnormalities in complete blood count, blood chemistry, and in liver, kidney, or pancreatic function tests [24].
3. In case of therapeutic failure, rescue treatment was applied with pentavalent antimonials, in which there are two possibilities, cure or therapeutic failure.
4. In the fourth state, the safety analysis of rescue treatment was added, including the three possibilities described in the second state.

No additional states were included for the subgroup that would require a second rescue treatment because their probability tended to be zero, nor was death taken as a final outcome due to the low mortality rate of cutaneous leishmaniasis in Colombia (0.006%).

### Model assumptions

The patients adhere to treatment (no abandonment), and all of them receive rescue treatment in case of therapeutic failure. The patients had not received other therapies for the disease 3 months before starting treatment. At the beginning of the treatment regimen, the patients did not present renal or hepatic failure, hematologic disorders, or comorbidities. The lesions had to be less than 10 per patient and at sites far from the nasal or oral mucosa, eyes, and anal or urogenital orifices. It should be clarified that these assumptions represent the characteristics of the majority of patients in the country (about 95% and 80% of the cases from Colombian Army’s leishmaniasis program and civilian population, respectively) and must be considered because the type of information recorded by SIVIGILA does not allow subgroup analysis.

### Cost-effectiveness analysis

The cost-effectiveness ratio is expressed in terms of the cost per DALY averted and patient cured in both alternatives. Incremental cost-effectiveness was used to estimate the cost per DALY averted or additional patient cured in the treatment with thermotherapy, compared to the use of Glucantime.

$$ \mathrm{Incremental}\  \mathrm{Cost}\  \mathrm{Effectiveness}\  \mathrm{Ratio}\ \left(\mathrm{ICER}\right)=\frac{\mathrm{C}}{\mathrm{E}}\ \frac{\mathrm{C}\mathrm{ost}\  \mathrm{of}\  \mathrm{Thermotherapy}\hbox{-} \mathrm{Cost}\  \mathrm{of}\  \mathrm{Glucantime}}{\mathrm{DALYs}\  \mathrm{averted}\  \mathrm{with}\  \mathrm{Thermotherapy}\hbox{-} \mathrm{DALYs}\  \mathrm{averted}\  \mathrm{with}\  \mathrm{Glucantime}} $$

### Sensitivity analysis

The uncertainty derived from the components of the decision model was evaluated using one-way deterministic analysis with tornado diagrams to evaluate the effect of changes in cure probabilities, costs, DALYs averted, and the number of patients cured. In addition, probabilistic sensitivity analysis was performed using Monte Carlo simulations including microsimulation of 1000 people, an acceptability curve for different thresholds or willingness to pay, and the net monetary benefit. The thresholds recommended by the WHO [23] were included, according to which a health technology is considered highly cost-effective for values less than a certain gross domestic product (GDP)/per capita (US$ 6056 for Colombia) and cost-effective for values less than three GDP/per capita.

For sensitivity analysis, it was assumed that probabilities have a β (beta) distribution in a narrow range from 0 to 1, whereas costs, DALYs averted, and the number of patients cured follows a γ (gamma) distribution that can take any value greater than zero. The limits of the confidence intervals of efficacy (cure and therapeutic failure) and safety (local and systemic adverse events) of previous clinical trials were taken as measures of variability [4, 10]. Regarding costs, a dispersion of 30% was taken, corresponding to the variability of tariffs used in contracting health services in Colombia; thus, the results presented are in line with the reality of payment and contracting of the country, where a relative variation of 20% was applied in the case of DALYs and patients cured.

## Results

**Fig. 1:**
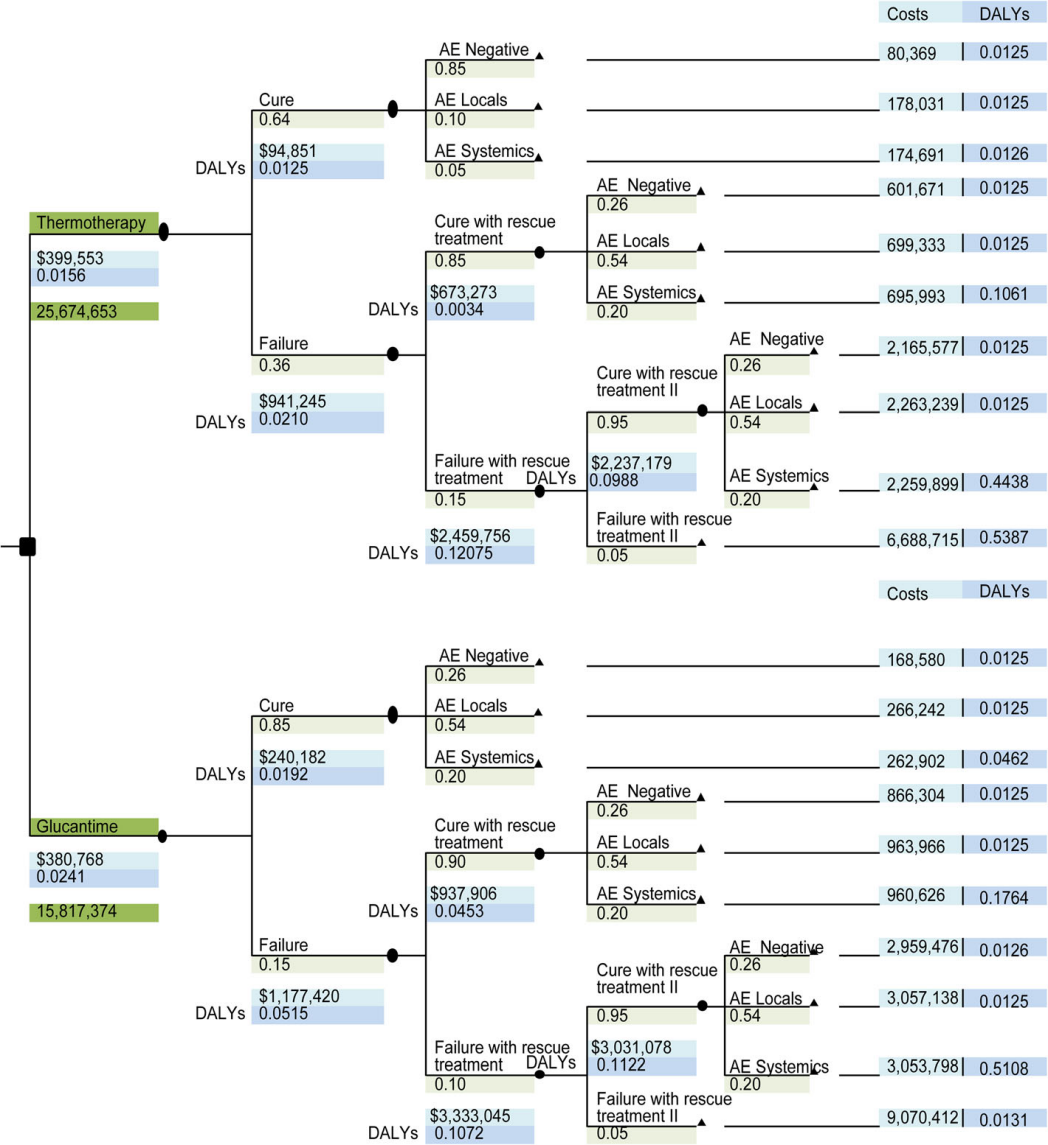
Decision tree for the analyses of the cost-effectiveness of thermotherapy, in comparison with Glucantime, in the treatment of cutaneous leishmaniasis (Costs and DALYs per patient)

Figure 1 shows decision tree for evaluating the cost-effectiveness of thermotherapy, compared to Glucantime, for the treatment of cutaneous leishmaniasis in Colombia (Costs and DALYs averted per treated patient), which shows the main health-state transition probabilities as well as the costs and adverse effects (DALYs).

Table 1 describes the costs that have been identified, measured, and evaluated. In patients receiving thermotherapy, the cost of the intervention is US$ 501,621; the handling of local adverse effects, US$ 20,255; and systemic events, US$ 8969. In patients who present therapeutic failure and receive rescue treatment based on pentavalent antimonials, the costs of their administration and use of diagnostic means to monitor local and systemic effects total US$ 300,053. The total cost of Glucantime administration in patients is US$ 2,731,276; the cost for the management of local effects is US$ 25,930; for systemic effects, US$ 32,324; and for therapeutic failures, US$ 406,298.

**Table 1:** Standard cost for thermotherapy and glucantime

	Item	Frequency	Cost per unit
Thermotherapy	ThermoMed (cryo)	1	14.04
	Medical Consultation	5	7.73
	Nurse (cures)	2	4.57
Glucantime	Glucantime	70	1.47
	Nurse (cures)	20	4.57
	Medical Consultation	4	7.73
	Creatinine	3	3.61
	BUN	3	2.58
	AST/ALT	3	11.21
	Amylase	3	4.19
	HLG	3	9.34
	ECG	1	10.56
Local effects	Myalgia	1	0.44
	Arthralgia	1	0.44
	Headache	1	0.44
	Abdominal Pain	1	1.10
	Fever	1	0.44
	Vomiting, Nausea, Anorexia, Diarrhea	1	1.70
	Infection of the Lesion	1	27.44
Systemic effects	Renal	1	6.18
	Pancreatic	1	4.19
	Hepatic	1	11.21
	Hematological	1	9.34

1 US Dollar = 3051 Colombian pesos (average value for 2016)

By disaggregating the costs for each of the model transition states, in the case of thermotherapy, it is found that the cost is US$ 142 per patient cured; if thermotherapy fails and rescue treatment is needed, then the cost amounts to US$ 655, and if a second rescue treatment is needed, then the cost would increase to US$ 2192. It should be noted that these values include the costs inherent to the management of adverse effects in each state. In patients treated with Glucantime, under the same conditions, the costs would be US$ 229, US$ 915, and US$ 2973, respectively.

Regarding effectiveness, in patients treated with thermotherapy, there were 149.0 DALYs that were caused to a great extent by the years of life lost in patients receiving a second rescue treatment with pentavalent antimonials, those having systemic adverse effects (patients in this group are at highest risk of dying, particularly if they are at the extremes of life or present diseases related to immunosuppression), and those with general disability that depend to a large extent on incident cases. In the Glucantime group, there were 195.6 DALYs explained to a great extent by the component of years of life lost by the group of patients with systemic adverse effects and the disability component due to the cumulative incidence (Table 2).

**Table 2:** DALYs of the clinical states of the decision model

State of the decision model	Thermotherapy				Glucantime			
	N	YLL	YLD	DALY	N	YLL	YLD	DALY
Cure after initial treatment								
Without adverse effects	4413	0.0	55.2	55.2	1793	0.0	22.4	22.4
Local effects	519	0.0	6.5	6.5	3724	0.0	46.6	46.6
Systemic effects	260	0.0	3.3	3.3	1379	46.5	17.2	63.7
Cure with rescue treatment I								
Without adverse effects	645	0.0	8.1	8.01	285	0.0	3.6	3.6
Local effects	1341	0.0	16.8	16.8	591	0.0	7.4	7.4
Systemic effects	497	0.0	6.2	6.2	219	35.9	2.7	38.6
Cure with rescue treatment II								
Without adverse effects	108	0.0	1.4	1.4	30	0.0	0.4	0.4
Local effects	225	0.0	2.8	2.8	62	0.0	0.8	0.8
Systemic effects	83	35.9	1.0	36.9	23	11.5	0.3	11.8
Failure with rescue treatment II								
Failure + Adverse effects	22	11.5	0.3	11.8	7	0.0	0.1	0.1

**Fig. 2:**
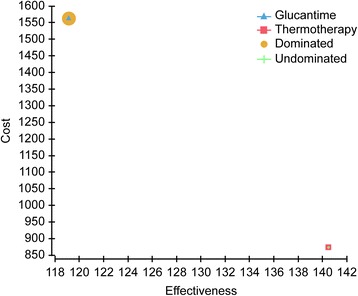
Cost-Effectiveness Analysis

Based on the DALYs shown in Table 2, the number of DALYs averted was calculated for each component of the model, which were then used for cost-effectiveness analysis; Thermotherapy generated 140.75 DALY averted and Glucantime 119.99 (Fig. 2. Cost-effectiveness plane (costs vs. DALYS averted) for thermotherapy and Glucantime). It should be noted that the behavior shown in Fig. 2 was similar to that found for the secondary outcome (number of patients cured).

The costs were US$ 290,187 for Thermotherapy and US$ 508,797 for Glucantime, which imply an incremental cost of treatment of US$ 218,610 with antimonial. In terms of effectiveness, Thermotherapy generated 140.75 DALY averted and it cured 4124 patients, reflecting an incremental efficiency, when it was compared with the Glucantime (20.76 DALY averted and 1892 patients cured). The cost-effectiveness of thermotherapy was US$ 2062 per DALY averted and US$ 70,4 per patient cured. For Glucantime, it was $ 4241 per DALY averted and US$ 84,6 per patient cured. The incremental cost-effectiveness ratio showed that thermotherapy would result in savings of US$ 10,530 for each additional DALY averted compared to Glucantime and that each additional patient cured would cost US$ 115,5 (Table 3).

**Table 3:** Cost-effectiveness analysis of thermotherapy vs. glucantime

Strategy	C	IC	E	IE	IC/IE	Dominance	CEA
DALYs averted							
Thermotherapy	290,187	–	140.75		–	–	2062
Glucantime	508,797	218,610	119.99	−20.76	−10,530	Dominated	4241
Patients Cured							
Thermotherapy	290,187	–	4124	–	–	–	70,4
Glucantime	508,797	218,610	6016	1892	115,5	–	84,6

*C* cost, *IC* incremental cost, *E* effectiveness, *IE* incremental effectiveness, *CEA* cost effectiveness average

**Fig. 3:**
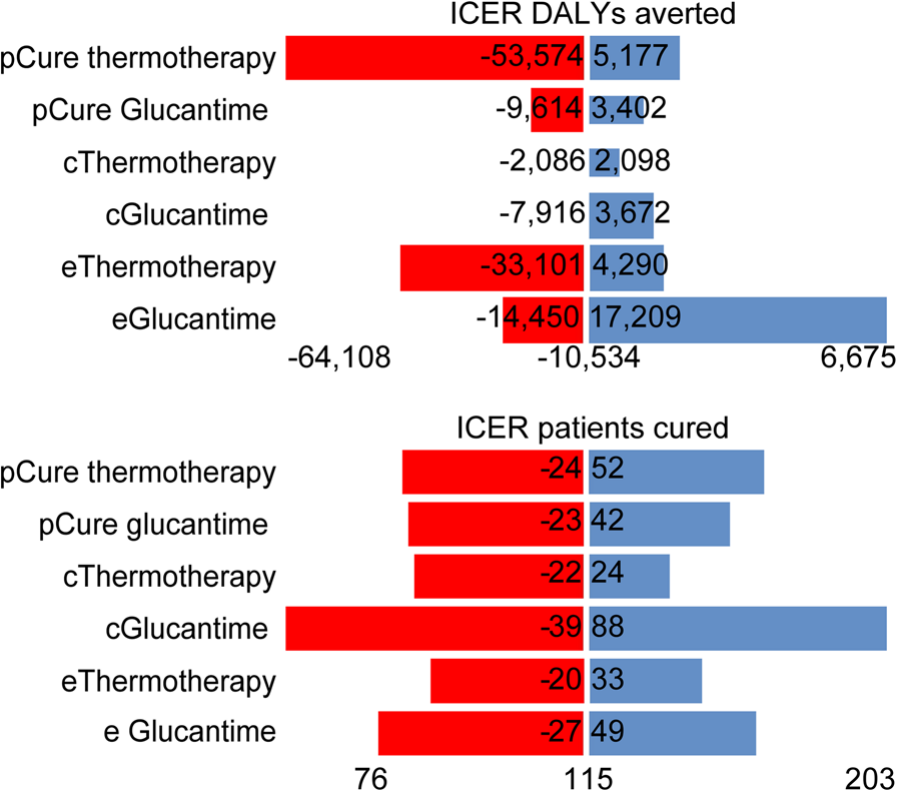
Deterministic analysis of sensitivity

The deterministic sensitivity analysis shows that the parameters that incorporate greater uncertainty in the incremental cost-effectiveness ratio are the change in the probability of cure with thermotherapy for DALYs averted and the cost of Glucantime for the number of patients cured. It is worth stating that thermotherapy would be cost-effective in all scenarios, considering its threshold of three times the Colombian GDP per capita. Given the highest effectiveness of thermotherapy, the ICER indicates that this treatment would avoid the expenses of US$ 64,108 per additional DALY averted, compared to the administration of Glucantime (the dominant strategy due to having lower cost and more effectiveness). Even if the highest effectiveness achieved by Glucantime were to be considered, it is estimated that thermotherapy would imply expenses of US$ 6675 per additional DALY averted; that is, even in the worse scenario, thermotherapy is highly cost effective (Fig. 3. Deterministic sensitivity analysis tornado diagram).

**Fig. 4:**
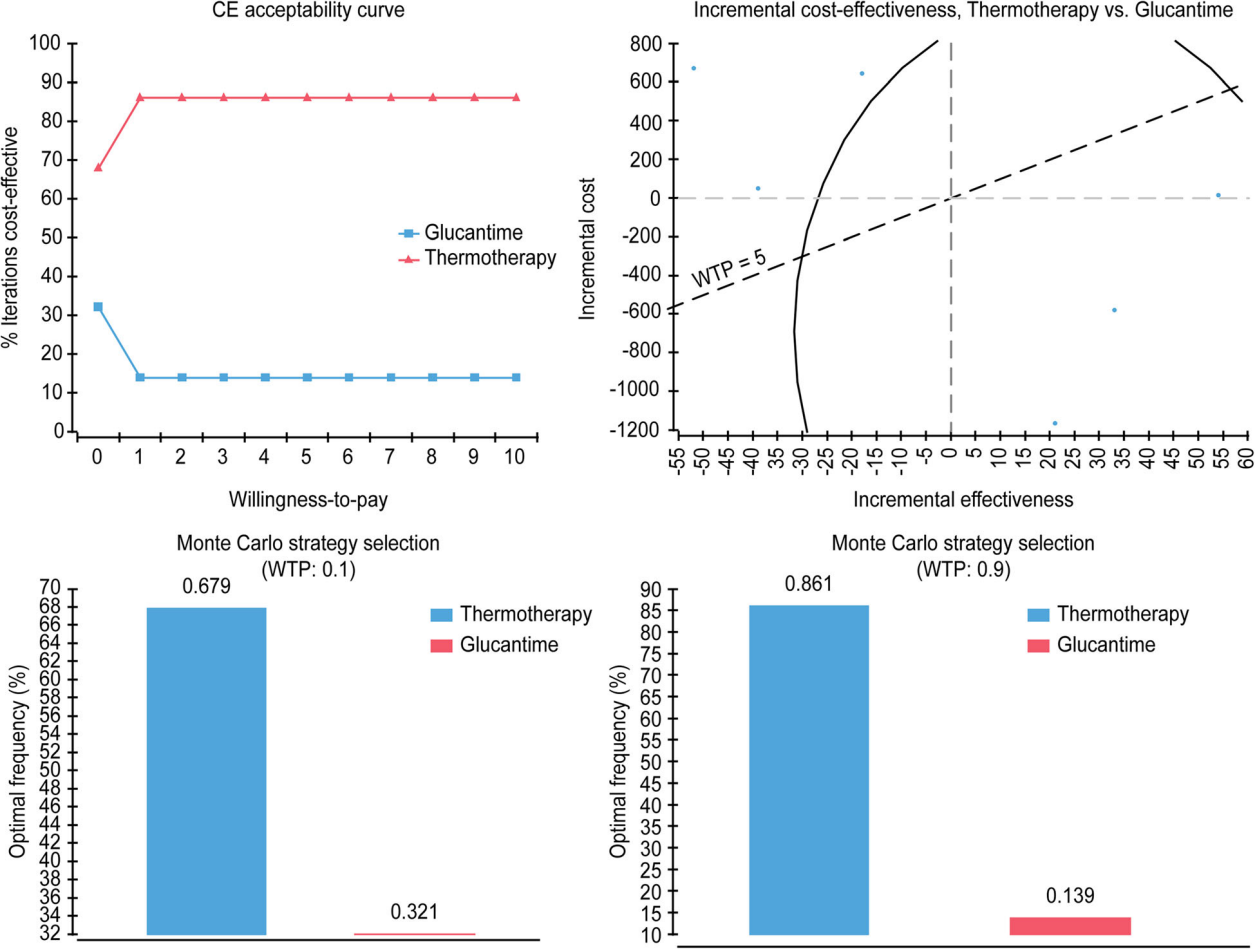
Probabilistc analysis of sensitivity. A. Monte Carlo Simulation. B. Incremental Cost-Effectiveness Scatter Plot. C. Optimum Monte Carlo Simulation (WTP: 0.1). D. Optimum Monte Carlo Simulation (WTP:0.9)

The acceptability curve from the probabilistic sensitivity analysis for DALYs averted shows that, independent of the willingness to pay, thermotherapy is more cost-effective, proving that it is a dominant strategy. The study of incremental cost-effectiveness shows that the majority of the simulated cases remain in quadrants I and IV. In the selection of the optimal strategy, with a willingness to pay of US$ 33 (100,000 Colombian pesos), thermotherapy is more cost-effective in 67.9% of the simulated cases, and if willingness to pay increases up to US$ 294 (900,000 Colombian pesos), then the same conclusion is reached in 86.1% of cases (Fig. 4. Sensitivity analysis by acceptability curve, incremental cost-effectiveness with threshold, and optimal strategy with 10,000 Monte Carlo simulations). For the number of patients cured, thermotherapy is identified as the optimal strategy in 68.2% of cases, using a threshold of US$ 300,000.

**Fig. 5:**
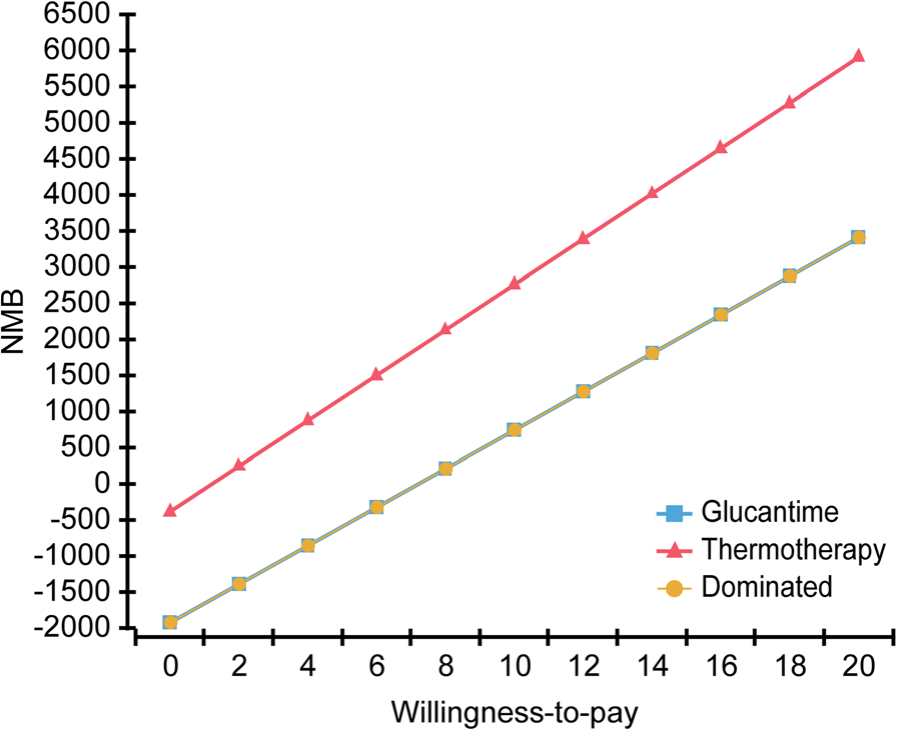
Net Monetary Benefit Cost-Effectiveness Analysis

Figure 5 shows net monetary benefit of thermotherapy and Glucantime for DALYs averted in cutaneous leishmaniasis according to different levels of willingness to pay. Even with different levels of willingness to pay, thermotherapy remains the strategy with the greatest net monetary benefit.

Finally, Table 4 shows the proportion of simulated cases in each of the quadrants of the cost effectiveness plan, highlighting that, for DALYs averted, thermotherapy is dominant in 67.9% of cases and cost-effective in 18.2% of cases, with an incremental cost-effectiveness ratio of less than US$ 300, whereas for the proportion of patients cured, it is cost-effective in 72% of cases.

**Table 4:** Probabilistic sensitivity analysis using 1000 Monte Carlo simulations

Component	Quadrant	IE	IC	ICER	% DALYs	% Cured
C1	IV	> 0	< 0	Superior	67.9	0.0
C2	I	> 0	> 0	< 1.0	18.2	72.0
C3	III	< 0	< 0	> 1.0	0.0	0.0
C4	I	> 0	> 0	> 1.0	0.0	12.8
C5	III	< 0	< 0	< 1.0	0.0	1.4
C6	II	< 0	> 0	Inferior	13.9	13.8
Indifferent	Origin	=0	=0	0/0	0.0	0.0

## Discussion

This the first study in the scientific literature that presents a cost-effectiveness analysis of thermotherapy for the treatment of cutaneous leishmaniasis, obtaining favorable results and a high level of evidence for patients in Colombia, given that the efficacy and safety data of comparative alternatives were taken from phase IIb and III controlled clinical trials with infected patients from this country. In addition, the findings from this study will have a high potential for extrapolation to populations with similar eco-epidemiological, clinical, and parasitological conditions.

The evidence presented adds to other previous publications, concluding that thermotherapy can be applied in patients with cutaneous leishmaniasis, to the extent that its therapeutic efficacy is similar to that of pentavalent antimonials, although with a lower proportion of adverse effects [4]. This is more relevant, considering some practical advantages of thermotherapy, such as shorter duration, better compliance, and the fact that it does not require paraclinical examinations and is able to be used in patients with renal, hepatic, or cardiac disorders, pregnant women, children, and other groups in which pentavalent antimonials or miltefosine are contraindicated [9, 22].

From an economic perspective, it should be considered that comprehensive economic evaluations of this disease are scarce, and none has evaluated local or physical treatments such as thermotherapy, which makes it difficult to compare the results found with those reported in previous publications. However, it is important to indicate the cost per DALY averted for other interventions implemented to treat this disease. In this sense, Reithinger’s study in Afghanistan reported that intralesional and intramuscular administration of antimonials generated a cost of US$ 27 per patient cured and US$ 1200 per DALY averted [20], which is significantly lower than the results of the present study and could be explained by the low cost of these drugs in Asian countries compared to South America.

For its part, the Orellano group in Argentina found an ICER of US$ 156 per DALY averted for an early diagnosis strategy and US$ 13,155 for the use of insecticide-impregnated clothing and curtains [17], which is higher than the alternative evaluated in this study and would constitute an important antecedent for designing subsequent cost-effectiveness studies that would allow the purchase of various disease interventions to determine whether vector control, timely diagnosis, conventional treatment, or new local and physical treatments are more or less cost-effective.

In two studies conducted in Colombia, it has been reported that the cost is US$ 345 per patient treated and cured with antimonials and US$ 15,215 per DALY averted during an outbreak of the disease [26]; in contrast, the evaluation of treatments included in the disease management guide in Colombia found that the incremental cost varies between US$ 10,000 and US$ 14,500 per DALY averted and between US$ 1000 and US$ 1500 per patient cured [15]. The differences found with respect to the current study could be based on three uncontrolled sources of uncertainty in the sensitivity analysis. The first difference would correspond to structural uncertainty, given that one of the studies cited used a Markov model in which infection relapse was not included as a therapeutic failure and the time horizon was higher than indicated to determine patient cure. The second would be a source of methodological uncertainty to the extent that some data from the Niño study were taken from phase II clinical trials or there were methodological quality problems. Finally, divergences between studies could also be due to the heterogeneity of the populations evaluated.

It should be borne in mind that the treatment of cutaneous leishmaniasis presents large cost variations related to the application protocol (intralesional or intramuscular), type of patient care, and health regimen, which should be considered in the uncertainty analysis. In addition, it is worth noting that studies that have recommended the use of pentavalent antimonials due to their low cost generally do not include the costs associated with the management of adverse effects [20, 25].

On the other hand, it is appropriate to state some of the reasons that have led to an increase in the use of cost-effectiveness analysis at present, i.e., the need (i) to prioritize funding for interventions, reduce health inequities, and address the well-being of future generations; (ii) to identify the best method of allocating health resources or optimizing the health budget; (iii) to avoid or transform inefficiencies that are present in many countries for the improvement of health states and to enact health policy, particularly in poor or middle-income countries, based on the costs and effects of different health interventions; and (v) to improve clinical practice guidelines [23].

Several limitations of this type of economic evaluation should also be considered, such as the type of cost items to be included, the extra costs incurred due to the years of life gained by an intervention, the variability in the manner in which interventions are implemented in different contexts or regions, and the assessment of the null effect when health processes are interrelated, among others [23]. The main limitation of this study is the fact that it was not possible to perform subgroup analysis because SIVIGILA records are deficient in the description of incident cases – according to some relevant characteristics for the estimation of cost-effectiveness – such as the infecting species and the number, size, type, and location of the lesions.

Consistent with the above, further studies should improve subgroup analysis and the integration of costs from the social perspective, considering that treatment with pentavalent antimonials generates higher costs related to transportation to the place of treatment, out-of-pocket costs to pay for outpatient services, indirect costs related to the loss of productive activities of patients and their families, etc. These costs are greater compared to thermotherapy, given that treatment with pentavalent antimonials requires more medical visits, bearing higher social costs related to the treatment itself and the management of adverse effects.

## Conclusion

Thermotherapy is a highly cost-effective strategy for the treatment of cutaneous leishmaniasis in Colombia. Its incorporation into clinical practice guidelines and public health programs could represent savings of approximately US$ 10,500 per additional DALY averted and the cost of US$ 115 per additional patient cured, compared to the use of Glucantime. This evidence adds to previous findings that have demonstrated the multiple benefits of this treatment alternative, such as better patient compliance, the simplicity of application, safety, and low costs. This is consistent with the objectives of the WHO, the Drugs for Neglected Diseases Initiative (DNDi), and other entities that seek to promote research on new drugs for this disease. This study will also help in guiding health policy actions for the management of this disease in Colombia and other countries with similar parasitological, clinical, and epidemiological patterns.
